# Synchronous Hartmann reversal and incisional hernia repair is associated with higher complication rate compared to a staged procedure

**DOI:** 10.1038/s41598-021-81064-3

**Published:** 2021-01-14

**Authors:** Y. Rudnicki, N. Horesh, Y. Lessing, V. Tverskov, A. Wachtel, M. Slavin, H. Tulchinsky, N. Wasserberg, E. Mavor, O. Zmora, S. Avital

**Affiliations:** 1 Department of Surgery B, Meir Medical Center, 4428164 Kfar Saba, Israel; 2 Department of General Surgery and Transplantations B, Sheba Medical Center, Ramat Gan, Israel; 3 Department of Surgery, Tel-Aviv Medical Center, Tel-Aviv, Israel; 4 Department of Surgery, Rabin Medical Center, Petah Tikva, Israel; 5 Department of Surgery, Kaplan Medical Center, Rehovot, Israel; 6 Affiliated to the Sackler School of Medicine, Tel Aviv University, Tel Aviv, Israel; 7 School of Medicine, Affiliated to the Hadassah-Hebrew University, Jerusalem, Israel

**Keywords:** Musculoskeletal system, Digestive signs and symptoms, Colonic diseases, Colon

## Abstract

Post operative ventral hernias are common following Hartmann's procedure. There is a debate whether hernia repair is safe when performed concomitantly with colostomy closure. In this study we aimed to evaluate the outcomes of synchronous Hartmann reversal (HR) with a hernia repair, compared to a staged procedure. A retrospective multi-center study was conducted, including all patients who underwent Hartmann’s procedure from January 2004 to July 2017 in 5 medical centers. Patient data included demographics, surgical data and post-operative outcome. Two hundred and seventy-four patients underwent colostomy reversal following Hartmann's procedure. In 107 patients (39%) a concomitant ventral hernia was reported during the Hartmann's reversal. Out of this cohort, 62 patients (58%) underwent hernia repair during follow-up. Thirty two patients (52%) underwent a synchronous hernia repair and 30 patients (48%) underwent hernia repair as a separate procedure. Post operative complication rate was significantly higher in the colostomy reversal with synchronous hernia repair group when compared to HR alone group (53% vs. 20%; *p* < 0.01; OR 4.5). In addition, severe complication rate (Clavien–Dindo score ≥ 3) was higher in the synchronous hernia repair group (25% vs. 7%). A tendency for higher hernia recurrence rate was noted in the synchronous group (56% vs. 40%). Median follow up time was 2.53 years (range 1–13.3 years). Synchronous colostomy closure and ventral hernia repair following Hartmann's procedure carries a significant risk for post operative complications, indicating that a staged procedure might be preferable.

## Introduction

Hartmann's procedure (HP) is regarded as a staged procedure, with restoration of bowel continuity in a second operation. However, multiple studies have shown that the rate of colostomy closure i.e. Hartmann reversal (HR) can be as low as 25 to 40%^1–3^. Hartmann's Reversal is a complex procedure associated with significant complication rates as high as 50%, including intra-abdominal septic complications, surgical site infections and other complications, most notably urinary tract and respiratory infections along with venous thromboembolism events^2–5^. Studies showed that up to 30% of patients will endure a severe complication that will require additional invasive intervention (Clavien–Dindo IIIa and above).

Postoperative ventral hernias (POVH), midline incisional hernia or parastomal hernia are common following Hartmann's procedure^6–11^, and may even be under-reported with rates ranging between 4 and 48%^9,12^. In patients undergoing a Hartmann reversal and have a POVH, there is a clinical dilemma whether to repair the abdominal wall defect synchronously with restoration of bowel continuity, or whether to stage the procedure into two separate major surgeries. The data on concomitant hernia repair during HR compared to separate operation is limited and preliminary data suggesting a higher complications rate with a synchronous repair compared to performing a HR alone^13^.

In a synchronous colostomy closure with incisional hernia repair, many surgeons would avoid using a synthetic implant, owing to concern of mesh infection and therefore choose to repair the hernias primarily, without abdominal wall mesh reinforcement. In the staged approach, surgery for the hernia repair will usually take place months after the patient has recovered from the HR, and most surgeons would prefer to use a synthetic mesh to lower hernia recurrence rates.

This study aims to report the prevalence of POVH after HP in patients undergoing HR, to compare the complications rate between a synchronous HR with POVH repair and HR alone and to assess hernia recurrence rate when comparing a synchronous versus a staged repair.

## Materials and methods

A retrospective-cohort multi-center study was conducted, including all patients who underwent Hartmann’s procedure from January 2004 to July 2017 in 5 medical tertiary referral centers in Central Israel. All centers had acute care surgery and colorectal surgery services. Patients’ data included demographic characteristics, surgical and clinical data including timing of the Hartmann’s reversal and timing of hernia repair (if any), surgical approach, length of stay, morbidity and mortality and post-operative outcome. Charlson comorbidity index (CCI) was used to classify patient comorbidities and for risk assessment^14^. The Clavien–Dindo classification of surgical complications scale was used to classify postoperative complications^15^. Primary outcome measures were the prevalence of post operative ventral hernia (POVH), midline and parastomal hernia rates after HP in patients undergoing HR, rate of complications after HR with or without synchronous hernia repair and the rate of hernia recurrence. Hernia recurrence was assessed by a combination of clinical evaluation based on symptoms and physical examination.

Each of the five participating centers local institutional review boards approved the study. All respective institutional review boards waived the need to sign an individual informed consent by each patient for this retrospective study.

Statistical analysis was performed using GraphPad Prism software version 8.0.1 (GraphPad Software, LLC, CA, USA). Continuous variables were expressed as mean ± standard deviation or medians and ranges, whereas discrete variables were expressed as numbers and percentages or frequencies. Data was compared using Fisher’s exact test and χ2 test to evaluate differences between qualitative variables and the Student’s t-test to compare quantitative variables. A *p* value of < 0.05 was considered statistically significant, similar to the analysis done in our previous report^2^.

## Results

Six hundred and forty patients that underwent Hartmann's Procedure were included in the database. Of those, only 274 patients (38.5%) underwent Hartmann's reversal (HR). 107 of these patients (39%) were reported to have a concomitant post operative ventral hernia (POVH) at the time of colostomy take down. POVH following Hartmann's procedure was associated with higher patients’ BMI (mean 28.2 ± 4.6 kg/m^2^ vs. 26.2 ± 4.4 kg/m^2^ in patients without POVH; *p* = 0.01) and had higher mean number of comorbidities (1.3 vs. 1.09 *p* = 0.04). There was no difference between the groups in American Anesthesiologist Society Association (ASA) score and The mean interval time that elapsed from the HP to the HR, 8.1 versus 7.2 months respectively (*p* = 0.56) (Table 1).

**Table 1:** Demographics of patients reported to have a postoperative ventral hernia (POVH) following a Hartmann's procedure (HP), while having their colostomy reversed compared to patients without a POVH.

	POVH following HP (n = 107)	No POVH following HP (n = 167)	*p* value
Gender F/M	57/50 (53 /47%)	78/89 (47/53%)	0.29
Age (year) [mean] (SD)	63.8 ± 13.42	61.3 ± 15.63	0.17
Body mass index [mean] (SD)	28.2 ± 4.61	26.25 ± 4.38	0.01
No. of comorbidities [mean] (SD)	1.3 ± 1.02	1.09 ± 1.11	0.04
Charlson score [mean] (SD)	1.96 ± 2.43	1.99 ± 2.66	0.94
Neoplastic cause to HP	26/107(24%)	46/167 (27.5%)	0.55
**ASA class; n (%)**			
I	13 (12%)	26 (16%)	0.79
II	54 (50.5%)	77 (46%)	
III	38 (35.5%)	62 (37%)	
IX	2 (2%)	2 (1%)	
Interval between HP to Hartmann reversal (months)	7.26	6.77	0.47

*ASA* American Society of Anesthesiologists, *POVH* post operative ventral hernia, *HP* Hartmann's procedure.

One hundred and seven patients were reported to have a concomitant post-operative ventral hernia at the time of colostomy reversal. Of those, 62 patients (58%) had their hernia repaired, synchronously or at a third later stage and all the rest had only skin closure with no fascia repair. Types of hernias reported included 65 midline incisional hernias (61%), 31 parastomal hernias (29%), 10 midline combined with parastomal hernias (9%) and one parastomal hernia with a laparoscopic port site hernia. Median follow up was 2.53 years (Table 2).

**Table 2:** Sub analysis of postoperative ventral hernia (POVH), following a Hartmann's procedure (HP), outlining types of hernias percentage of hernia repair.

Operative findings	n	%	*p* value
No. of patients reported with POVH during HR	107/274	39	
No. of hernias repaired	62/107	58	
No. of female with hernia repair	30/57	53	0.247
No. of male with hernia repair	32/50	64	
**Types of hernias reported**			
Midline incisional hernia	65	61	
Parastomal hernia	31	29	
Midline incisional + parastomal hernia	10	9	
Parastomal + laparoscopic port site hernias	1	0.93	
Median follow up time and range (years)	2.53	(1–13.3)	

*POVH* post operative ventral hernia, *HP* Hartmann's procedure, *HR* Hartmann reversal, *d* days, *y* years.

### Perioperative outcome—comparison between synchronous Hartmann reversal combined with hernia repair to Hartmann reversal alone

**Figure 1:**
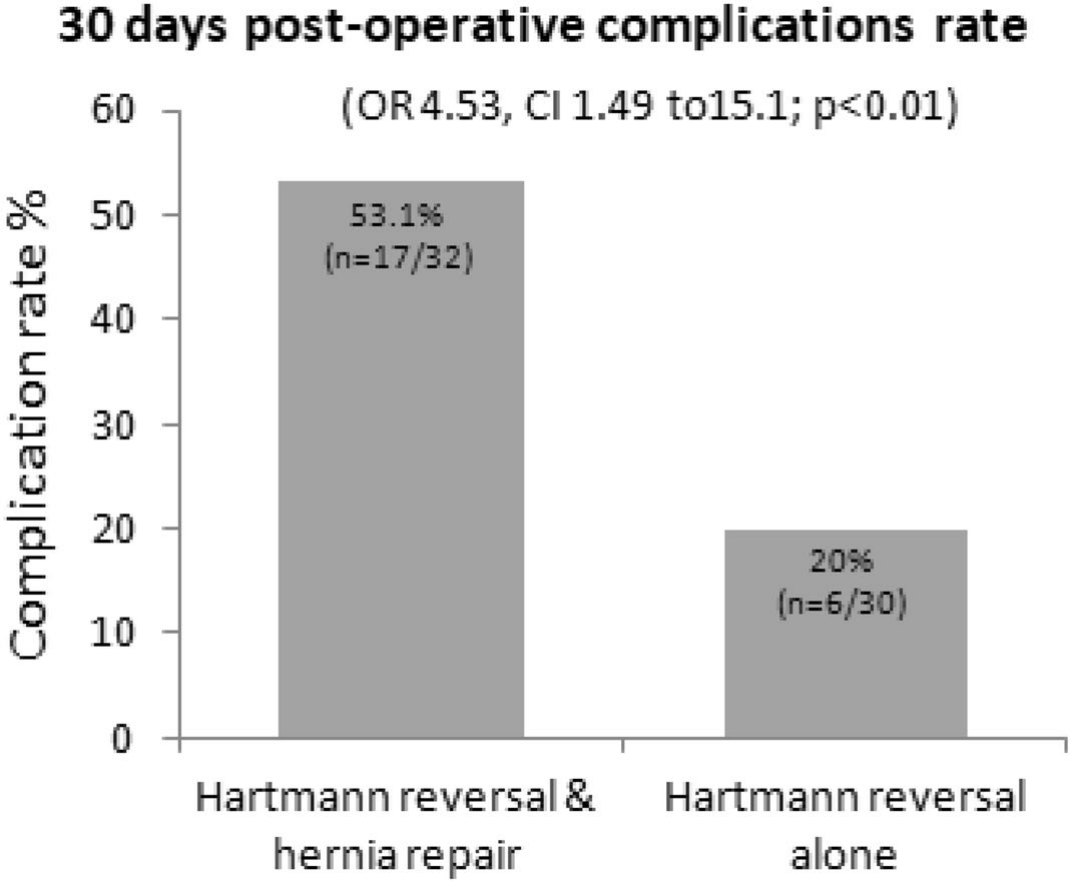
Thirty days post-operative overall complication rate following synchronous Hartmann reversal and hernia repair compared to Hartmann reversal alone (without hernia repair).

Of the 62 patients who had their hernia repaired, 32 patients (52%) underwent a synchronous repair while 30 patients (48%) underwent reversal of Hartman and had their hernia repaired in a third separate operation. There was a lower mean BMI in the synchronous group compared to the staged group (26.6 ± 4.4 kg/m^2^ vs. 30.4 ± 4.6 kg/m^2^, *p* = 0.033). Thirty days post-operative overall complication rate following synchronous Hartmann reversal and hernia repair was 53% (17 patients), compared to 20% (6 patients) after HR alone without hernia repair (OR 4.5, CI 1.49 to 15.1; *p* < 0.01), (Fig. 1). Increased rate of complication in the synchronous group was evident for surgical site infections, small bowel obstruction, bleeding and wound complications. In the synchronous repair group eight patients (25%) had a wound dehiscence and failure of the hernia repair while still hospitalized. When comparing the severity of complications, Eight patients (25%) in the synchronous repair group had Clavien–Dindo complication grade of IIIa or above compared to two patients (7%) in the HR alone group (*p* = 0.08) with one patient in the synchronous group that died following multiple laparotomies after a small bowel obstruction with bowel perforation and sepsis. Post operative complications are detailed in Table 3. The mean length of stay following the HR was 13.5 days for the synchronous group versus 10.3 days in the HR alone group (*p* = 0.12) and the mean interval time that passed between the HP and the HR was 8.1 versus 7.2 months respectively (*p* = 0.56).

**Table 3:** Description of perioperative outcome—patients with POVH following HP, which underwent Hartmann reversal with POVH repair compared to patients that underwent Hartmann reversal alone.

Complications and time intervals	Hartmann reversal with POVH repair (n = 32)	Hartmann reversal without POVH repair (n = 30)	*p* value
30 days postoperative complications	17 (53%)	6 (20%)	0.009
SSI	6 (19%)	2 (7%)	0.2
Bleeding	3 (9%)	1 (3%)	0.6
SBO	4 (12.5%)	0	0.1
Anastomotic leak	0	1 (3%)	0.5
Incarcerated POVH	0	1 (3%)	0.5
Wound dehiscence	8 (25%)	1 (3%)	0.027
Clavien–Dindo I-II	9 (28%)	4 (13%)	0.2
Clavien–Dindo ≥ IIIA	8 (25%)	2 (7%)	0.08
LOS [mean] (SD) (days)	13.5 ± 10	10.3 ± 5	0.12
Interval between HP to HR (months)	8.1	7.2	0.56

*POVH* post operative ventral hernia, *SSI* surgical site infection, *SBO* small bowel obstruction, *Clavien–Dindo* the Clavien–Dindo classification of surgical complications, *LOS* Length of stay, *HP* Hartmann's Procedure, *HR* Hartmann reversal.

### Postoperative outcome—comparison between synchronous Hartmann reversal combined with hernia repair to staged hernia repair after Hartmann reversal

**Figure 2:**
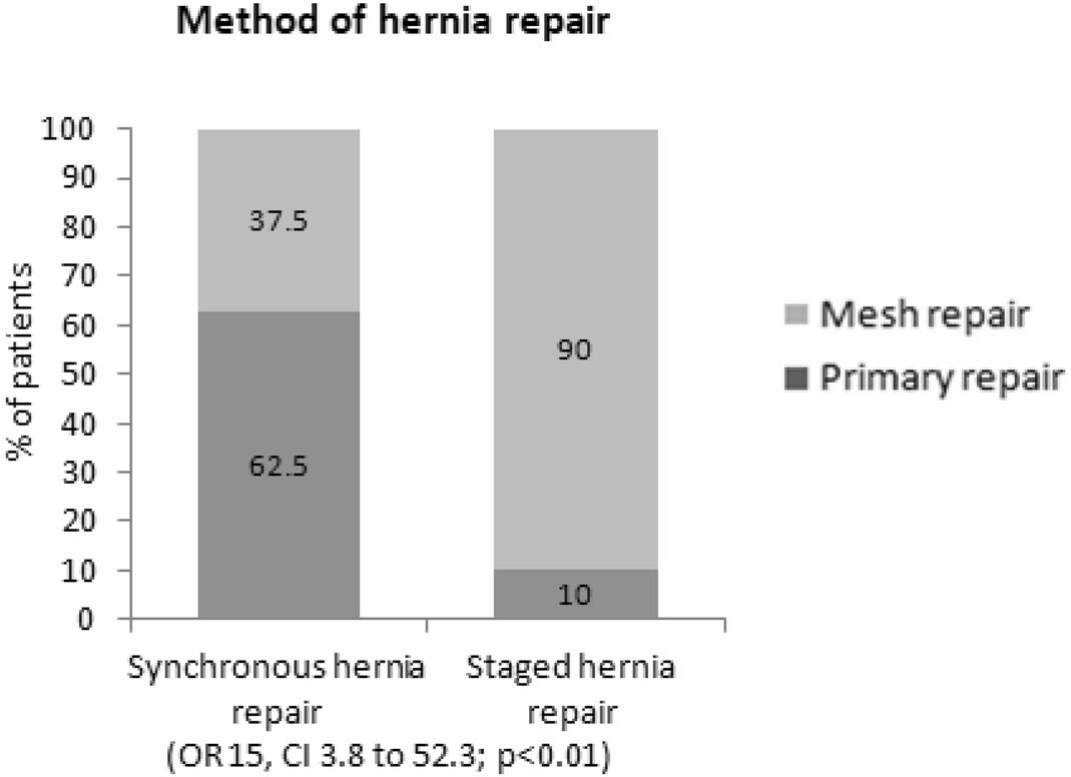
Method of hernia repair (primary repair or tension free mesh repair) in the synchronous hernia repair group compared to the staged hernia repair group.

The 32 patients (52%) in the synchronous hernia repair group were compared to the same 30 patients (48%) in the Hartman reversal alone group after they had the staged hernia repair in a third separate operation. The hernia types in each group are detailed in Table 4. The method of hernia repair was significantly different with a primary repair (i.e. suture repair) in 62.5% (20 patients) in the synchronous group compared to 10% (three patients) in the staged group (*p* < 0.01) with a correlating tension free mesh repair in 90% (27 patients) of the staged group compared to 37.5% (12 patients) of the synchronous group (OR 15, CI 3.8 to 52.3; *p* < 0.01), (Fig. 2). The repair method and sutures used were chosen based on the center and surgeon preference. There were no reports of any mesh being treated with antibiotics pre-insertion or prophylactic antibiotics for extended period with mesh use, other than perioperative antibiotics upon induction.

**Table 4:** Comparison between synchronous hernia repair group and staged hernia repair group—detailing types of hernias, methods of repair and recurrence rate.

	Synchronous hernia repair (n = 32)	Staged hernia repair (n = 30)	*p* value
Gender F/M	15/17	15/15	> 0.9
Age (year), mean ± SD	64 ± 15.22	62.8 ± 14.1	0.76
Body mass index, mean ± SD	26.6 ± 4.4	30.4 ± 4.6	0.03
**Type of hernia**			
Midline POVH, n (%)	18 (56%)	19 (63%)	0.38
Parastomal hernia, n (%)	11 (34%)	6 (20%)	
Midline + parastomal, n (%)	3 (9%)	5 (17%)	
**Method of hernia repair**			
Primary repair, n (%)	20 (62.5%)	3 (10%)	< 0.001
Mesh repair, n (%)	12 (37.5%)	27 (90%)	
Synthetic mesh repair, n (%)	8 (25%)	23 (77%)	
Biological mesh repair, n (%)	4 (12.5%)	4 (13%)	
POVH Recurrence, n (%)	18 (56%)	12 (40%)	0.2

*POVH* post operative ventral hernia.

Out of the twelve patients in the synchronous group treated with a mesh repair, eight patients had a synthetic mesh inserted and four patients had a biological mesh inserted. Out of the 8 synthetic mesh patients, two had a hernia recurrence and no SSI's. Out of the 4 biological mesh patients, two had SSI's and the mesh was removed. Out of the 27 patients in the staged group treated with a mesh repair, 23 patients had a synthetic mesh inserted and four patients had a biological mesh inserted.

Median follow up was 2.53 years with a range of 1 to 13.3 years. Thirty (48%) Out of the 62 patients that had a synchronous or a staged hernia repair suffered from hernia recurrence. Eighteen patients (60%) had an additional surgery for hernia repair. Subdivision of the hernia recurrence showed that 18 patients of the synchronous repair group had a recurrence compared to 12 patients in the staged repair group (*p* = 0.2). Nine patients (50%) with hernia recurrence in the synchronous group had another attempt of hernia repair compared with nine patients (75%) in the staged group. Data regarding hernia repair and recurrence is detailed in Table 4.

The overall rate of laparoscopic HR was 22%. The rate of laparoscopic HR did not differ between patients with or without a POVH (23% vs. 21% respectively). Only 3 patients in the synchronous group underwent laparoscopic HR compared to 6 patients in the staged group, 9% versus 20% (*p* = 0.9).

After the separate hernia repair in the staged repair group, 3 patients had a SSI and were treated with antibiotics alone, and there was one patient that had an infected hematoma that was drained and he was treated with antibiotics and a Vacuum-Assisted Closure of a wound (VAC). Overall, a 13% complication rate for the separate hernia repair stage, with a cumulative complication rate of the staged group of 33%. All four patients had a synthetic mesh repair.

### Human and animal rights

This study has been approved by the appropriate institutional research committee of each participating medical centers, and has been performed in accordance with the ethical standards as laid down in the 1964 Declaration of Helsinki and its later amendments or comparable ethical standards.

### Study protocol and informed consent

The study protocol was approved by the institutional research committee (IRB-Helsinki committee Meir Medical Center, Kfar Saba, Israel). For this type of study (retrospective in nature), formal consent is not required. An exemption from informed consent for this study was given by the institutional research committees (IRB-Helsinki committee Meir Medical Center, Kfar Saba, Israel), as the data were retrospectively retrieved from an existing prospectively and routinely collected database.

## Discussion

Hartmann reversal with regain of colonic continuity can be a challenging procedure. As such, it is not surprising that more than half of the patients never have their stoma closed, and those that undergo colostomy closure are usually younger, have a benign disease and less significant comorbidities. The prevalence of post Hartmann ventral hernia is high compared to other types of abdominal surgeries^9^, with 39% of patients in this study having a ventral hernia at the time of HR. Some would even say that there is an underreporting of parastomal hernias, basing this suspicion on the prevalence of parastomal hernias in retrospective compared to prospective studies^16^. It is possible that some small parastomal hernia go unreported since they are primarily repaired when closing the colostomy's defect in the fascia.

The general condition of patients undergoing Hartmann’s procedure, which often have septic conditions and malnutrition, most likely contributes to the high rate of postoperative ventral hernia, and patients’ characteristics such as BMI and a higher number of comorbidities were found in our study to correlate with an increased risk. These findings are in line with those described in previous reports^17,18^.

The overall incidence of incisional hernia in any kind of midline laparotomy is 11–20% and is associated with potentially life threatening conditions including bowel incarceration or strangulation^6–8,10,11^. The overall reported incidence of parastomal hernia after any kind of stoma can be as high as half of patients. The specific prevalence of parastomal hernia following end colostomy may be as high as 48%, correlating with the findings is this study. It is unclear whether the location of the stoma in regards to the rectus muscle, the size of the trephine or fascial fixation have any influenced on the occurrence of parastomal hernia^9^. Parastomal hernias are also thought to be an independent risk factor for incisional hernia with a seven times greater risk of occurrence compared to patients without parastomal hernia^12^.

Both Hartmann’s colostomy and postoperative ventral hernia impact patients’ quality-of-life and are the main indication for reversal and repair of the hernia. Concomitant repair of both conditions may gain remedy for two illnesses in one operation. Both Hartmann’s reversal and repair of postoperative ventral hernia are usually major abdominal surgeries, with significant recovery period, and it makes sense to save the patient a second major procedure if possible. The fact that 42% of the patients who were diagnosed with ventral hernia at the time of closure of Hartmann never had their hernia repaired, probably owing to the magnitude and risk, further emphasizes the logic of the option to alleviate both conditions in one procedure. Specific nutritional parameters were not recorded in this study. However, all Hartmann reversals and hernia operations were performed in elective settings months after the patients recovered from their acute illness. Thus we assume that, in general, all patients were in a satisfactory nutritional status.

On the other hand, Hartmann reversal is a clean contaminated procedure, and there is a concern using synthetic meshes in such a condition. Indeed, in our study most of the concomitant group patients were treated with a primary hernia repair and most of the staged group treated with a mesh repair. The reported post operative hernia recurrence after primary repair (direct tissue repair) ranges from 46 to 100% compared to the lower rate of mesh repair recurrence of 0 to 33.3% in various reports^9,19–21^. There are no firm guidelines to clearly support concomitant or stage repair, and evidence-based literature is limited. The results of our study show that despite having lower BMI, concomitant repair is associated with significantly higher rate of complication rate, severe complications, and a trend towards increased rate of failure of the hernia repair. It should be emphasized that almost half of the synchronous repairs failed while still hospitalized. It could be that the cause for the major morbidity in the synchronous group was likely due to a flawed repair method. This is possible since most of these repairs were done using a primary suture repair method, not necessarily done by abdominal wall specialist and it might be that in the mind of the colorectal surgeon reversing the stoma, the hernia repair is not the main focus of the procedure, which may allow for a less meticulous repair technique.

Although this study focuses on the time point of the surgeons' decision on a synchronous or a staged hernia repair, one should consider the complications rate of the separate hernia repair operation in the staged approach. We believe that repairing a hernia in a separate stage following a Hartman reversal should be evaluated and discussed like any other post-operative hernia repair following any abdominal surgery.

The main limitations of this study lay in its retrospective nature and hence the lack of a standard decision process on which patients should have their hernia repaired, what is the optimal timing of reversal /repair and what surgical approach. There were only 62 patients in both arms combined and they differ in the types of hernias and treatment approach, most notably the use of mesh or not. Another potential drawback of the retrospective nature of the study is the fact that diagnosis of recurrent hernia is based on review of medical charts and not on prospective physical examination. Never the less, there was a high rate of recurrence in the staged group which is difficult to explain and might be due to their surgical history of two prior surgeries and a challenged abdominal wall. There was data missing regarding the synchronous hernia repair method, which may overlook an inadequate repair technique, as most repairs were done using a primary suture repair, and if were done using a mesh, preferably by an abdominal wall specialist, may have a had better results. Adding to that, the anatomic plane into which mesh was placed or the ratio of mesh size to defect size is also missing and may impede the ability to extrapolate these results to a wider population. A median follow up of over two and a half years should allow an adequate diagnosis of hernia recurrence, and all five medical centers cover most of the central part of Israel, allowing cross-referencing medical information of patients, narrowing the chance for patients lost for follow-up.

With the lack of prospective studies and clear guidelines for the timing and treatment of post Hartmann's hernias, this cohort may provide insight into the decision-making process of POVH repair, suggesting that the drawbacks of concomitant repair may outweigh its benefits. Further prospective studies are needed to consolidate these results and are being planned.

## Conclusions

Post operative incisional hernia after a Hartmann procedure is common. According to this data, the added risks of a synchronous hernia repair while reversing the colostomy and a trend towards lower success rate, suggests that a synchronous repair might be inferior to a staged approach. Having said that, a synchronous repair is still a viable approach, but if chosen, it is prudent to plan the repair in advance in order to avoid an inferior repair method. An abdominal wall specialist can be consulted and utilizing a mesh may be considered, as even separate hernia repair in these kinds of conditions may also have complications.
